# The Association of Natural Elements and Trail Use by Adults

**Published:** 2011-12-15

**Authors:** Anna E. Price, Julian A. Reed, Steve P. Hooker

**Affiliations:** Department of Physical Therapy and Human Movement Sciences, Sacred Heart University; Department of Health Sciences, Furman University; School of Nutrition and Health Promotion, Arizona State University. At the time of this study, Dr Hooker was affiliated with the Arnold School of Public Health at the University of South Carolina

## Abstract

We used the System for Observing Play and Recreation in Communities to examine the association between variations in natural elements (ie, season, weather, and temperature) and adults' use of a rail-trail in South Carolina (2006-2009). Most (62%) of the 4,468 trail users observed were walkers; 38% were observed participating in vigorous physical activity. Adults were most often observed using the trail during the summer (32%), sunny weather (78%), and moderate temperatures (54%). When promoting trail use among adults, natural elements should be considered.

## Objective

Studies indicate that variations in season, weather, and temperature may influence physical activity (PA) behavior (1,2) such as trail use. The Community Preventive Services Task Force recommends the creation of, or enhanced access to, places such as greenway trails (3,4) to increase PA and improve people's physical fitness (5). However, few studies concerning these variations have been conducted; researchers have called for additional studies that examine associations between PA behavior and natural elements (1,4). We examined the effects of seasonal differences and variations in weather and temperature on adults' trail use.

## Methods

Adult trail use (ie, PA behavior while using the trail) was examined on a 2-mile urban rail-trail (ie, a railroad track that was converted to a trail) in Spartanburg, South Carolina, from fall 2006 through spring 2009. This trail connects the downtown business district and more rural parts of the county and is located between 2 historic residential neighborhoods that include people with diverse socioeconomic and demographic characteristics (6). Trained observers used the 7-day version of the System for Observing Play and Recreation in Communities (SOPARC) (7) to assess trail user demographics and behaviors (determined by trained observers) and to record variations in season, weather, and temperature.

Undergraduate students were trained as trail observers (details provided elsewhere [6]). Test–retest correlation coefficients (*r*) for observations between observers were greater than 0.90 for each variable for all observers. Observers visited the trail alone and made observations for 10 minutes at 6 access points on the trail in the morning (7:30 am), at noon (12:30 pm), and during the afternoon (3:30 pm) and evening (6:00 pm). Observers coded the PA behavior of people using the trail as walking (ie, at a casual pace) or engaging in vigorous PA (eg, jogging, cycling) (7). People who were observed engaging in sedentary activities were excluded from the analysis. The observers also coded the season, weather, and temperature during each observation period.

Only observations of adults aged 21 to 59 years (SOPARC protocol [7]) using the trail were examined in this study. The University of South Carolina and Furman University institutional review boards approved the study. Statistics were calculated by using SAS version 9.1 (SAS Institute Inc, Cary, North Carolina). Logistic regression models were used to compare adults observed walking versus engaging in vigorous PA by season, weather, and temperature. Sex, ethnicity, and time of day were controlled for in all regression analyses. Significance was set at P <.05.

## Results

During 16 quarterly observation periods of the trail from 2006 through 2009, 4,468 observations of adults engaging in PA were recorded. Trail users were mostly white (68%) and men (51%) (Table 1). Most (62%) trail users were walkers, and 38% were observed participating in vigorous activity (data not shown).

The volume of adults using the trail was greatest during the summer (32%), sunny weather (78%), and moderate temperatures (54%). We compared observations of walking versus engaging in vigorous activity while using the trail by season, weather, and temperature adjusting for sex, ethnicity, and time of day (Table 2). Adults were more likely to walk during the summer than during the winter or fall; they were less likely to walk in cloudy weather than in sunny weather and during high temperatures than during moderate temperatures. Adults were more likely to engage in vigorous PA in the fall and winter than in the summer, during cloudy weather than during sunny weather, and at low temperatures than at moderate temperatures.

## Discussion

The volume of adults observed using the trail was greatest during the summer, when the weather was sunny and the temperature was moderate. These and similar findings from previous studies (8,9) suggest that promoting initial trail use among adults may be ideal when the weather is sunny and temperature is moderate. A positive experience using trails in favorable conditions may strengthen adults' intentions and self-efficacy for continued trail use (10).

We identified significant differences in adults' PA intensity by variations in season, weather, and temperature. Although adults were more often observed walking during favorable weather conditions, adults engaging in vigorous PA were more often observed during less favorable conditions. One explanation may be that adults who usually engage in vigorous PA are more committed to their PA routine and use the trail even in less favorable weather conditions. Health professionals and trail planners may consider providing environmental supports such as water fountains and covered seating areas and safety information for people using the trail in less desirable weather conditions. Additional research may examine the potential effect of such environmental supports or information on year-round use of trails.

There are limitations to this study. First, the observers used their own judgment to determine the demographic characteristics of trail users, which may have resulted in some error. Furthermore, the trail was 2 miles long; results may not generalize to longer trails (eg, 50-mile trails). Causal inferences about the associations between adults' trail use and natural elements cannot be made because of the cross-sectional nature of the study. However, the large number of users observed, valid and reliable objective measures used, and multiple seasons of data collection lend strength to the findings.

In conclusion, the identified significant relationships between adults' trail use and season, weather, and temperature may be useful for professionals planning and managing recreational trails or promoting trail use among adults.

## Figures and Tables

**Table 1:** Adults' Observed Physical Activity Behavior on a South Carolina Rail-Trail, by Seasons and Demographic Variables, 2006-2009^a^

**Variable**	Total (n = 4,468), No. (%)	Walking (n = 2,768), No. (%)	Vigorous Physical Activity (n = 1,700), No. (%)
**Season**			
Fall	984 (21.5)	533 (19.3)	414 (24.4)
Winter	907 (19.9)	521 (18.8)	372 (21.9)
Spring	1,229 (26.9)	817 (29.5)	391 (23.0)
Summer	1,448 (31.7)	897 (32.4)	523 (30.7)
**Temperature**			
Low (60°F)	1,123 (24.9)	721 (26.4)	383 (22.9)
Moderate (61°-80°F)	2,410 (53.5)	1,516 (55.4)	844 (50.6)
High (81°F)	971 (21.6)	499 (18.2)	442 (26.5)
**Weather**			
Sunny	3,463 (77.8)	2,171 (79.6)	1,206 (74.2)
Cloudy	828 (18.6)	450 (16.5)	366 (22.5)
Rainy	160 (3.6)	105 (3.9)	54 (3.3)
**Time**			
Morning	1,128 (24.7)	731 (26.4)	370 (21.8)
Noon	984 (21.5)	580 (21.0)	383 (22.5)
Afternoon	1,099 (24.1)	664 (24.0)	418 (24.6)
Evening	1,357 (29.7)	793 (28.6)	529 (31.1)
**Year**			
2006	1,194 (26.1)	656 (23.7)	527 (31.0)
2007	606 (13.3)	365 (13.2)	227 (13.4)
2008	1,517 (33.2)	925 (33.4)	534 (31.4)
2009	1,251 (27.4)	822 (29.7)	412 (24.2)
**Sex**			
Female	2,222 (48.6)	1,645 (59.4)	533 (13.4)
Male	2,346 (51.4)	1,123 (40.6)	1,167 (68.6)
**Ethnicity**			
White	3,099 (67.8)	1,681 (60.7)	1,371 (80.7)
Nonwhite	1,469 (32.2)	1,087 (39.3)	329 (19.3)

a Because some data is missing, the total number of responses for each variable category may not add up to the total number of observations listed.

**Table 2:** Adults' Physical Activity Behavior on a South Carolina Rail-Trail by Weather, Season, and Temperature, 2006-2009 (n=4,468)^a^

**Variable**	Walking vs Vigorous Physical Activity	
	OR (95% CI)	*P* Value
**Season**		
Fall	0.85 (0.69-1.06)	.006
Winter	0.76 (0.62-0.94)	
Spring	1.00 (0.89-1.32)	
Summer	1 [Reference]	
**Weather**		
Cloudy	0.62 (0.52-0.74)	<.001
Rainy	0.95 (0.66-1.36)	
Sunny	1 [Reference]	
**Temperature**		
Low (60°F)	1.12 (0.92-1.36)	.001
High (81°F)	0.70 (0.58-0.86)	
Moderate (61°-80°F)	1 [Reference]	

Abbreviations: OR, odds ratio; CI, confidence interval.

a The model was adjusted for sex, ethnicity, and time period.
